# Targeted antibiotic and dietary approaches in managing small intestinal bacterial overgrowth across irritable bowel syndrome subtypes

**DOI:** 10.1007/s11739-026-04418-7

**Published:** 2026-06-17

**Authors:** Yousra Iftequar, Prakhar Bajpai, Rajvi Dav, Kaza Sri Harshitha, Chandan C, Rakshit Goel, Sneha Chimkar, Rishi Chowdhary

**Affiliations:** 1 VRK Women’s Medical College, Hyderabad, Telangana 500075 India; 2 Jawaharlal Nehru Medical College, Belagavi, Karnataka 590010 India; 3 Shri Guru Ram Rai Institute of Medical and Health Sciences, Dehradun, Uttarakhand 248001 India; 4 Malla Reddy Medical College for Women, Hyderabad, Telangana 500055 India; 5 Karnataka Medical College and Research Institute, Hubballi, 580021 India; 6 Acharya Shri Chander College of Medical Sciences, Jammu, 180019 India; 7 Topiwala National Medical College, Mumbai, Maharashtra 400008 India; 8 Department of Medicine, MetroHealth Medical Center, Cleveland, OH 44109 USA

**Keywords:** Small intestinal bacterial overgrowth, Irritable bowel syndrome, Rifaximin, Low-FODMAP diet

## Abstract

**Introduction:**

Irritable bowel syndrome (IBS) is a prevalent functional disorder, comprising constipation-predominant (IBS-C), diarrhea-predominant (IBS-D), and mixed (IBS-M) subtypes. Emerging research has shown that the pathophysiological influences of SIBO are significant in IBS, particularly in IBS-D. Variations in therapeutic responsiveness and diagnostic paradigms across IBS subtypes necessitate a tailored, subtype-specific management framework.

**Methods:**

A review of identification strategies, SIBO subtype-specific prevalence, and treatment outcomes was supplemented through controlled randomized trials and observational studies. The treatment outcomes were evaluated in relation to SIBO and within the frameworks of rifaximin, neomycin, probiotics, and dietary modulation.

**Results:**

The prevalence of SIBO is notably greater in IBS patients, particularly in those with IBS-D, with figures estimating ranges from 30 to 60% depending upon the diagnostic methods and cutoff thresholds. Though limited in its sensitivity and standardization, breath testing, particularly glucose hydrogen/methane tests, remains the most widely accessible diagnostic technique. Methane-predominant SIBO in IBS-C does respond better to rifaximin–neomycin combinations, but rifaximin provides strong symptom relief in IBS-D. Significant gaps persist, however, with the absence of longitudinal outcome measurements and standardization of IBS diagnostic criteria. In order to improve the care for IBS patients, the strategies targeting precision medicine with microbiota profiling and algorithm-driven personalized therapies must be accorded priority.

**Conclusions:**

SIBO is increasingly recognized as a potential contributor in a subset of patients with IBS, with rifaximin being most beneficial for IBS-D, particularly when utilized in addition to dietary measures. IBS-C and IBS-M require individualized, multimodal therapy, and future studies should direct efforts toward subtype-specific, precision medicine treatments for improved long-term outcomes.

## Introduction

**Fig. 1:**
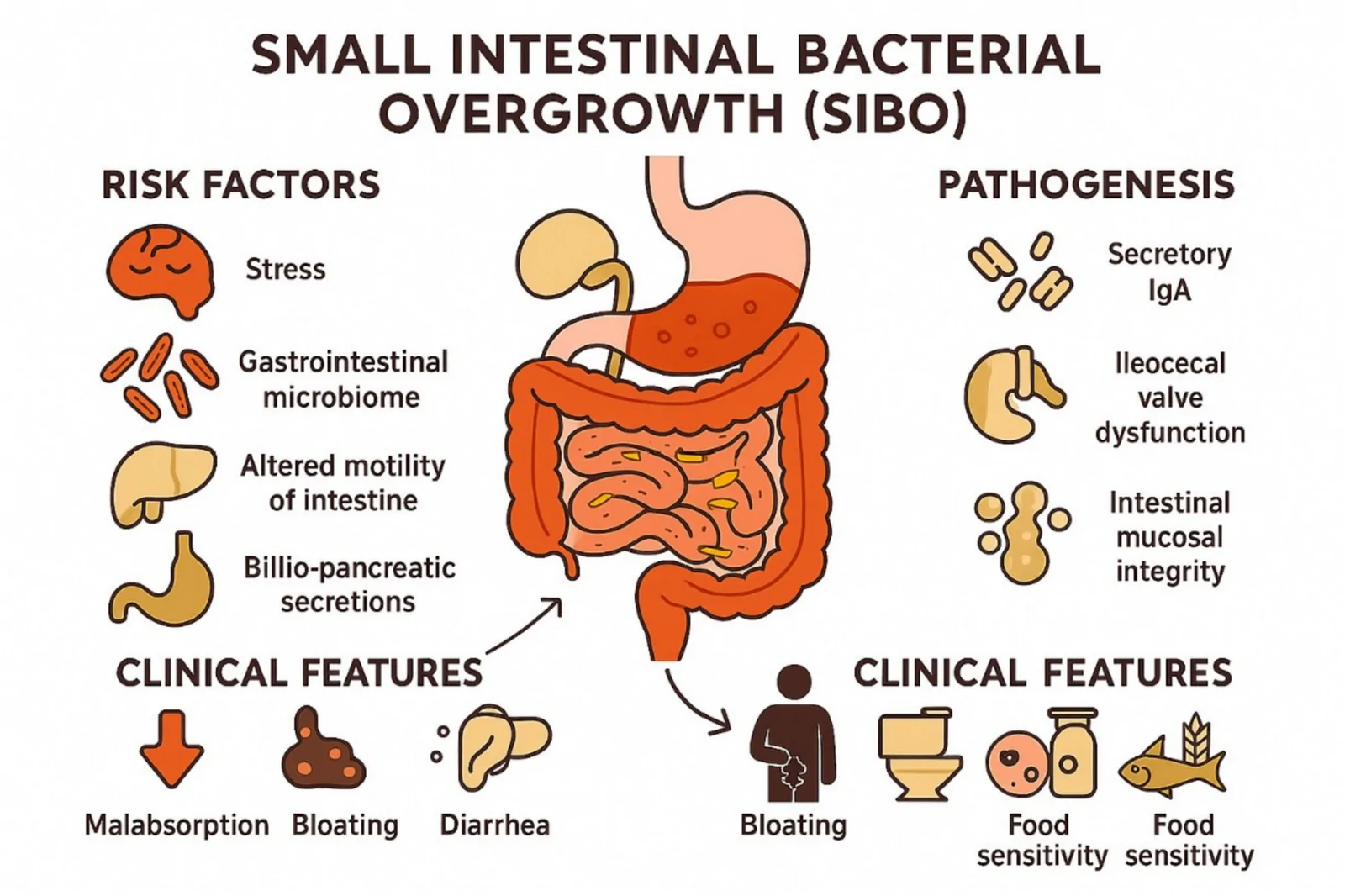
SIBO Pathogenesis and clinical manifestations [1, 2]. This figure summarizes the multifactorial pathogenesis of SIBO, including motility disorders, immune dysfunction, and mucosal integrity loss, leading to symptoms, such as bloating, malabsorption, and food sensitivities

Irritable bowel syndrome (IBS) has been referred to by various names over time, such as mucous colitis and chronic nonspecific enteropathy. It is a functional disorder that severely impacts an individual's daily life and professional activities. It causes abdominal pain and disrupts bowel habits. It often affects young adults and women, often coexisting with other functional gastrointestinal disorders (FGIDs), causing a substantial burden on personal well-being and the broader economy [1]. Global estimated prevalence of IBS is estimated between 10 to 25%, though regional differences are significant, with South America reporting higher rates (17–21%), while the lowest are reported in South Asia (7–9%). The pathogenesis of IBS is multifactorial, involving several interconnected mechanisms. These include altered gastrointestinal motility, heightened visceral sensitivity, post-infectious changes, disruptions in brain-gut communication, imbalances in gut microbiota, bacterial overgrowth, food intolerances, impaired carbohydrate absorption, and often coexists with low-grade intestinal inflammation. imposing both personal and economic burden [2](Fig. 1). Currently, the diagnosis, classification, and management of IBS are guided by the Rome Foundation, whose expert working groups have released four successive editions (Table 1). The approach to diagnosing IBS has significantly advanced, with the Rome IV criteria now serving as the gold standard for clinical evaluation. Unlike earlier practices, IBS is no longer regarded merely as a diagnosis of exclusion even though it shares symptoms with many other gastrointestinal conditions. Management strategies are tailored according to the specific subtype of IBS, namely, IBS-C, IBS-D, mixed-type IBS (IBS-M), and unclassified IBS (IBS-U) [2, 3].

**Table 1:** Rome III and IV criteria for diagnosis of IBS [3]

Rome III	Characteristics	Rome IV	Characteristics
IBS-C (IBS with constipation)	Hard/lumpy stools > 25% and loose/watery stools < 25% of bowel movements	IBS-C (IBS with predominant constipation)	More than 25% of bowel movements are hard or lumpy (Bristol types 1–2), and less than 25% are loose or watery (types 6–7)
IBS-D (IBS with diarrhea)	Loose/watery stools > 25% and hard/lumpy stools < 25% of bowel movements	IBS-D (IBS with predominant diarrhea)	More than 25% of bowel movements are loose or watery (Bristol types 6–7), and less than 25% are hard or lumpy (types 1–2)
IBS-M (mixed IBS)	Hard/lumpy stools > 25% and loose/watery stools > 25% of bowel movements	IBS-M (IBS with mixed bowel habits)	More than 25% of bowel movements are hard or lumpy (Bristol types 1–2) and loose or watery (Bristol types 6–7)
IBS-U (unsubtyped IBS)	Insufficient abnormality of stool consistency to meet criteria for IBS-C, D, M	IBS-U (IBS unclassified)	Patients meet the criteria for IBS, but their stool patterns don't fit any of the specific subtypes above

Recent research has explored a wide range of contributing factors in IBS, including gut microbiota imbalances (dysbiosis). Small intestinal bacterial overgrowth (SIBO) is classically defined as >10^5^ colony-forming units (CFU) per milliliter of small bowel content, which remains the gold standard despite its invasiveness [4, 5]. In clinical and research practice, non-invasive breath tests are more commonly employed. According to the North American Consensus, a positive test is defined as a rise in hydrogen of > 20 ppm within 90 min or methane > 10 ppm at any time. Methane-predominant cases are now increasingly referred to as intestinal methanogen overgrowth (IMO) rather than SIBO. SIBO is a known outcome of dysbiosis within the small intestine and is commonly observed in individuals with IBS. It is characterized by symptoms, such as abdominal bloating, flatulence, and changes in bowel habits. It often extends beyond the gastrointestinal tract, potentially leading to extraintestinal manifestations and complications [6]. A meta-analysis of 25 studies found that the prevalence of SIBO was significantly higher in patients with IBS compared to controls, 31.0% (95% CI 29.4–32.6%) versus 20.9% (95% CI 19.5–22.2%). The overall odds ratio (OR) for SIBO in IBS patients was 3.7 (95% CI 2.3–6.0). Subgroup analysis showed a higher association in studies with healthy controls (OR = 4.9, 95% CI 2.8–8.6) than in those with non-IBS disease controls (OR = 1.3, 95% CI 0.6–3.3).

A subset of IBS patients has been found to have SIBO. This has shifted the way we understand IBS, from being viewed primarily as a condition rooted in psychological stress or emotional triggers to recognizing that, for many, there may be a more biological or organic cause. This evolving perspective marks a significant turning point in how we approach both the diagnosis and treatment of IBS [4, 7].

## Methodology

### Study design

This manuscript is a narrative review synthesizing available evidence on the role of small intestinal bacterial overgrowth (SIBO) in irritable bowel syndrome (IBS), with a focus on targeted antibiotic and dietary interventions stratified by IBS subtype. A narrative approach was deliberately chosen given the substantial heterogeneity of study designs, diagnostic criteria, and outcome measures in this field, which precludes reliable meta-analytic pooling. The review aims to provide a clinically oriented synthesis, contextualize therapeutic evidence within IBS subtypes, and identify key knowledge gaps to inform future research priorities.

### Literature search strategy

A comprehensive literature search was conducted on PubMed/MEDLINE, Cochrane, Embase, and Scopus. The search was restricted to English language publications from January 2000 to January 2026.

The following search terms were used in various combinations using Boolean operators (AND, OR): *"Small intestinal bacterial overgrowth", "SIBO", "intestinal methanogen overgrowth*", "irritable *bowel syndrome*", *"IBS", "IBS-D", "IBS-C", "IBS-M", "rifaximin", "neomycin", "antibiotic", "low-FODMAP", "gut microbiota", "dysbiosis", and "breath test".*

Reference lists of all included articles and relevant systematic reviews were hand-searched for additional eligible studies not identified through the database search.

### Inclusion and exclusion criteria

Studies were included if they met the following criteria:

- Original research articles, systematic reviews, meta-analyses, and RCTs reporting on the prevalence, diagnosis, or treatment of SIBO in adult IBS populations
- Studies using Rome II, III, or IV diagnostic criteria for IBS subtype classification
- Studies employing standardized SIBO diagnostic methods (lactulose or glucose hydrogen/methane breath testing, or jejunal aspirate culture)
- Studies reporting outcomes related to antibiotic therapy (particularly rifaximin and/or neomycin), probiotic use, or dietary interventions (low-FODMAP, elemental, or specific carbohydrate diets)
- Pathophysiological and mechanistic studies relevant to the SIBO-IBS relationship.

Studies were excluded if they:

- Were published in languages other than English.
- Reported only on structural or post-surgical SIBO without relevance to functional IBS.
- Were case reports or editorials without original data.
- Lacked clear diagnostic criteria for either SIBO or IBS.

### Study selection and data synthesis

Titles and abstracts were independently screened by two reviewers (Y.I. and P.B.). Full-text articles were retrieved for all potentially eligible studies, and final inclusion decisions were made by consensus. In the event of disagreement, a third reviewer (R.C.) was consulted. Given the narrative design, no formal risk-of-bias assessment tool was applied.

### Pathophysiological link between SIBO and IBS

Although the exact mechanisms behind IBS are unclear, emerging research suggests that dysbiosis may play a role in triggering symptoms, at least in a portion of affected individuals. In SIBO, excess bacteria in the small intestine ferment dietary carbohydrates, leading to the production of gasses, such as hydrogen, methane, and carbon dioxide. This can contribute to common symptoms like abdominal bloating, distension, flatulence, and discomfort or pain [4].

In the pathophysiology of SIBO, we distinguish between 2 potential clinical manifestations of SIBO:

1. SIBO as a cause of maldigestion/malabsorption: The clinical presentation can be related to the effects of contaminating organisms on host morphology or function that, in turn, result in the clinical consequences typically associated with SIBO, such as steatorrhea, diarrhea, protein-losing enteropathy, and/or specific deficiency states [8].
2. SIBO is associated with symptoms or clinical entities in the absence of evidence of maldigestion/malabsorption (e.g., SIBO in association with IBS), and where the pathophysiological linkage with SIBO is less clearly defined [7].

The relationship between SIBO and IBS is still debated and far from conclusive. There is no strong evidence that SIBO develops after the onset of IBS symptoms, nor is there a consistent dose–response relationship between the concentration of bacteria in the small intestine and the severity of IBS symptoms. Studies exploring this connection have produced mixed and often contradictory results [1]. Moreover, many effective treatments for IBS, such as dietary changes, antispasmodics, and psychological therapies, do not target SIBO, yet they often have a lower number needed to treat (NNT) than antibiotics like rifaximin. This challenges the notion that SIBO is a central or primary driver of IBS. Additionally, IBS does not exhibit the clinical behavior typical of an infectious disease, making a purely microbial cause unlikely. While the brain–gut axis theory fits well with our understanding of gut function and behavior, the bacterial hypothesis does not have similar support. Therefore, although bacteria and SIBO may contribute to symptoms in a subset of IBS patients, they are unlikely to represent the sole or primary cause, and a definitive causal relationship remains unestablished [1, 4].

A particularly informative subgroup in this context is post-infectious IBS (PI-IBS), defined as new-onset IBS meeting Rome IV criteria following acute infectious gastroenteritis, with a pooled prevalence of approximately 10–11.5% and IBS-D and IBS-M as the predominant phenotypes. PI-IBS represents one of the most well supported linking gut infection, dysbiosis, and IBS with post-infectious alterations in microbiota composition, driving low-grade mucosal inflammation, increased intestinal permeability, impaired neuromuscular function, and gut-brain axis dysregulation, collectively creating conditions favorable to SIBO development. Notably, *Clostridioides difficile* infection (CDI) has emerged as a significant and underappreciated precipitant; *Iacob *et al*.* found that PI-IBS developed in 57% of confirmed CDI patients versus 25% of controls, with 90% of hospitalized CDI patients developing PI-IBS (RR = 6.1), underscoring the role of infection severity in post-infectious gut dysfunction [9]. Given that dysbiosis is central to PI-IBS pathophysiology, microbiome-targeted approaches may hold particular promise in this subgroup, as supported by Fusco et al., who demonstrated a 100% treatment response with microbiome-profiling-guided modulation versus 55% with standard therapy (p < 0.0001), highlighting PI-IBS as a valuable model for precision microbiome-directed therapy in IBS [10].

IBS subtyping increases the chances of finding unique biological processes behind each IBS type. Patients with IBS-C have a different gut microbial profile. They tend to harbor higher levels of *methanogens*, a group of microbes from the Archaea domain, unlike those with IBS-D, who typically lack these organisms. These methanogens convert dietary and endogenous carbohydrates into methane gas. Once thought to be biologically inactive, methane is now recognized for its role in slowing intestinal transit and even acting like a neurotransmitter. This has opened new doors for pharmacological interventions aimed at targeting methanogenesis to manage symptoms in IBS-C. The strong link between methane and IBS not only highlights a promising therapeutic avenue but also underscores the importance of studying this relationship in greater depth [11].

The gut–brain axis (GBA) is a bidirectional neural pathway that consists of the central nervous system, the autonomic nervous system, and the enteric nervous system. It also includes the neuroendocrine-immune regulation, stress response pathways, and visceral sensory conduction [12]. The interaction between the gut microbiota and the gut–brain axis is known as the gut microbiota-gut-brain axis. Dysbiosis in the microbiome and the ensuing dysfunction of the GBA cause changes in intestinal motility, permeability and secretion, which contribute to cellular alteration of the enteroendocrine system and visceral hypersensitivity. Evidence indicates that microbial dysbiosis, marked by instability and reduced diversity, frequently occurs in post-infectious IBS and often coexists with small intestinal bacterial overgrowth [13, 14].

SIBO as a causative factor for IBS has been explored in several studies, as in patients with IBS, the frequency of SIBO was 4–78%, while in healthy controls it was 1–40% [15]. A meta-analysis [16] exploring this association, analyzing data from 3192 patients with IBS and 3320 controls, demonstrated that the prevalence of SIBO in patients with IBS was significantly higher than in controls (odds ratio = 3.7). However, the overall quality of the evidence was low. The effectiveness of rifaximin in reducing symptoms of IBS has been cited as supporting evidence for a link between SIBO and IBS. However, since IBS may arise from abnormal fermentation occurring in the colon rather than the small intestine, it is also possible that rifaximin improves IBS symptoms by targeting harmful colonic bacteria rather than those in the small intestine [17].

The disease modifier hypothesis proposes that SIBO arises as a downstream consequence of IBS pathophysiology, specifically the dysmotility and altered transit, which collectively create a permissive environment for bacterial overgrowth. Under this model, SIBO amplifies and perpetuates symptoms in a subset of patients without being their root cause. This is supported by the high symptom recurrence rates following antibiotic therapy without correction of underlying motility dysfunction and by the observation that many effective IBS treatments, such as dietary modification, antispasmodics, and gut-directed psychotherapy, achieve meaningful symptom relief without targeting SIBO at all [7].

The epiphenomenon hypothesis represents the most skeptical position, arguing that SIBO as currently defined in the IBS literature is largely a diagnostic artifact. In a 2024 clinical practice update jointly endorsed by the European Society of Neurogastroenterology and Motility (ESNM) and the American Neurogastroenterology and Motility Society (ANMS), Kashyap et al. concluded that after two decades, the SIBO-IBS hypothesis remains unproven and that its most significant unintended consequence has been the widespread use of unreliable breath tests and resultant injudicious antibiotic prescribing. From this perspective, positive breath tests in IBS patients reflect accelerated orocecal transit and colonic fermentation rather than true small intestinal overgrowth, and rifaximin's benefit may operate through colonic microbiome modulation entirely independent of SIBO [18].

### Diagnostic approaches for SIBO in IBS patients

Accurate diagnosis of SIBO remains a cornerstone of IBS treatment, particularly in symptomatic subtypes, such as IBS-D and IBS-M. Hydrogen and methane breath tests (HBTs), particularly with lactulose or glucose as substrates, are the most commonly used diagnostic tests. They are not invasive, are easily accessible, and allow retesting, and thus are the viable option in both clinical and research settings [19, 20]. However, it is important to recognize at the outset that no currently available breath test achieves both high sensitivity and high specificity simultaneously, and the clinical utility of these tests in IBS populations remains a subject of considerable debate.

Lactulose hydrogen breath testing (LHBT) is also largely employed due to its convenience and potential for detecting distal SIBO. The accuracy of its diagnosis is, however, substantially limited by high false-positive rates arising from rapid orocecal transit, a physiological feature particularly prevalent in IBS-D, which can produce early colonic hydrogen peaks that are misattributed to small intestinal bacterial activity [8]. Reported sensitivity for LHBT ranges from approximately 52–70%, with notably low specificity, undermining its reliability as a standalone diagnostic tool.

Glucose breath testing (GBT), by contrast, benefits from near-complete proximal absorption of glucose, conferring higher specificity; however, this same property renders it insensitive to distal SIBO, producing false-negative results when bacterial overgrowth is located beyond the proximal small intestine. Sensitivity for GBT varies widely in the literature, ranging from as low as 20% to as high as 93%, reflecting the profound impact of patient preparation, substrate dose, and sampling protocols on test outcomes [8]. A study by *Rana *et al. demonstrated a higher SIBO detection rate with LHBT compared to GBT in IBS patients, but found GBT to be more discriminatory between IBS patients and controls, suggesting that the optimal test choice depends on clinical context, rather than a universal preference for one modality [8].

Methane measurement during breath testing adds another diagnostic layer. Methane positivity has been associated with IBS-C, reinforcing the value of dual H₂ and CH₄ analysis in stratifying IBS subtypes [21]. However, the clinical interpretation of methane readings is not without complexity. Research by *Wei H *et al*.* [22] highlighted the influence of dietary fat intake on breath methane levels, suggesting that both physiological and behavioral factors may confound test outcomes.

Critically, *Harvie *et al. [21] questioned the validity of the commonly used ≥ 20 ppm methane threshold for identifying carbohydrate malabsorption or SIBO, citing marked intra- and inter-individual variability in readings during testing, which is a concern that has yet to be resolved through standardized protocols.

Taken together, the limitations of breath testing across all substrates fall into three broad and clinically significant categories:

1. False positives/negatives: Accelerated intestinal transit (common in IBS-D) inflates false-positive rates on LHBT, while GBT systematically underdiagnoses distal SIBO [8]. These opposing error patterns mean that neither test alone adequately captures the full spectrum of SIBO in IBS.
2. Standardization gaps: Significant heterogeneity exists across studies and clinical sites in carbohydrate doses, sampling time, and diagnostic points (e.g., hydrogen rise thresholds of 10 ppm vs. 20 ppm) reduce reproducibility and comparability of results [8, 23].
3. Modest and inconsistent correlation with clinical outcomes: Perhaps most importantly, normalization of breath test results following treatment shows only a weak association with symptom resolution. This dissociation is illustrated by rifaximin trials, where symptomatic improvement was observed even in patients without confirmed breath test normalization, with SIBO eradication rates ranging from only 32–68% [19, 24].

**Fig. 2:**
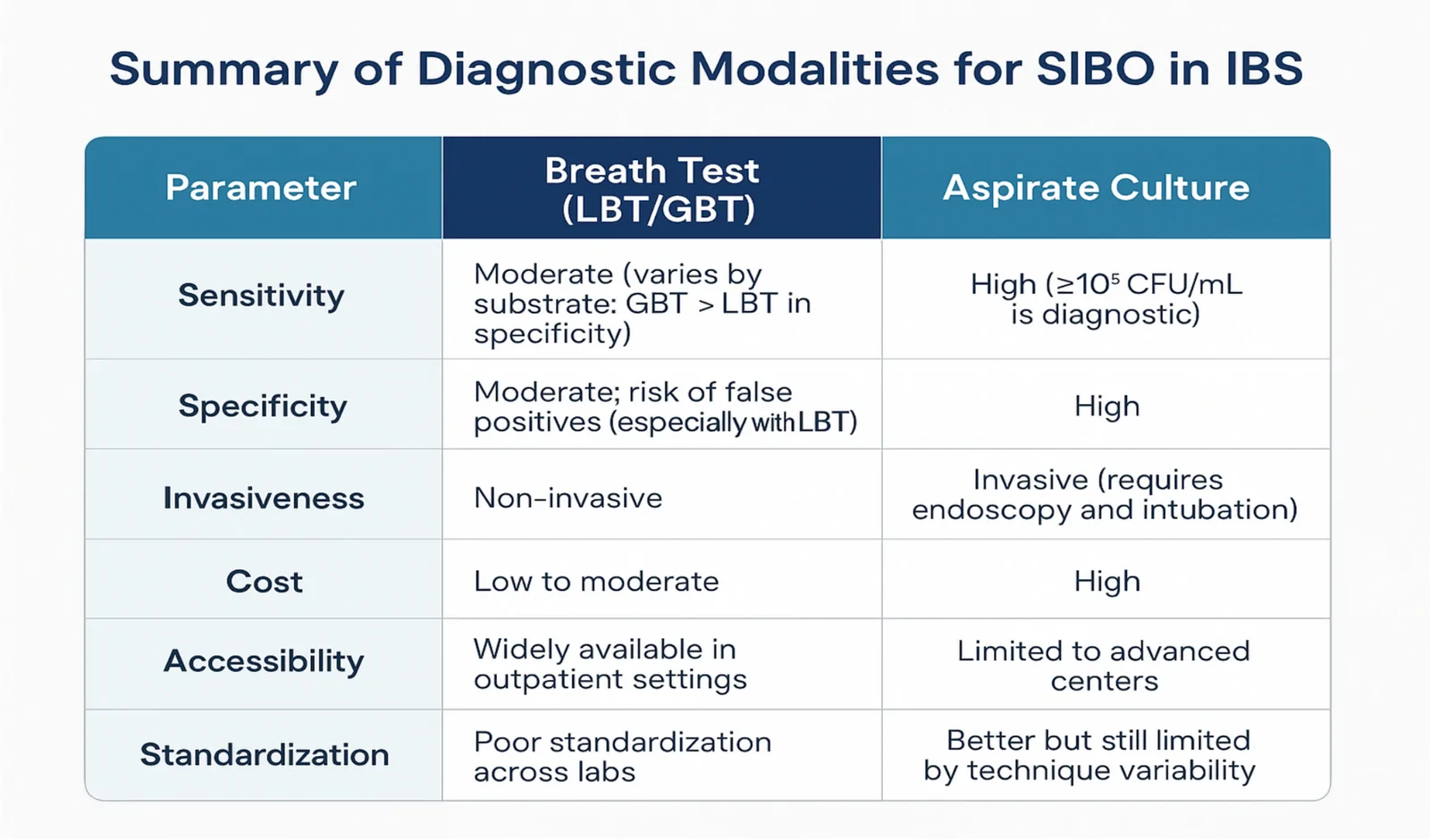
Comparison of diagnostic modalities for detecting SIBO in IBS [8, 18, 21]. Diagnostic modalities for SIBO in IBS: breath tests are non-invasive and accessible but limited by moderate accuracy and poor standardization, whereas aspirate culture provides higher sensitivity and specificity at the expense of invasiveness, cost, and availability

Small bowel aspirate and culture remain the gold standard for the diagnosis of SIBO due to their ability to directly measure the bacterial load (≥ 10^5^ CFU/mL), with sensitivity and specificity exceeding 90%. However, its invasive nature, procedural cost, risk of oropharyngeal contamination during sampling, and limited clinical availability restrict its use to research settings or cases where breath testing is indeterminate and clinical suspicion remains high [25] [Fig. 2].

Given these diagnostic uncertainties, the practical clinical approach continues to rely on integration of symptom-based Rome IV criteria with targeted breath testing, interpreted within the context of the individual patient's IBS subtype and symptom burden. Testing is most useful in the following contexts:

- 1) IBS-D/IBS-M: SIBO prevalence of up to 60% in patients with prominent bloating, flatulence, or maldigestion, making breath testing a reasonable first step before empiric antibiotic therapy [24, 26].
- 2) IBS-C: Methane-dominant SIBO is commonly identified; breath test results can guide the choice between antibiotic regimens and probiotic strategies [8].
- 3) Refractory cases: Post-treatment breath testing may help identify persistent or recurrent SIBO in non-responders, though the clinical significance of test normalization without symptom improvement must be carefully weighed [27].

As an adjunct to standard breath testing, methane detection, dietary assessment, and emerging microbiota profiling approaches [28] may enhance diagnostic specificity in selected patients. Moving forward, the field requires not only more standardized breath test protocols but also validation of novel biomarkers (including hydrogen sulfide), and microbiome-based signatures that may offer greater mechanistic precision in diagnosing and subtyping SIBO within the heterogeneous IBS population [Table 2].

**Table 2:** Review of Literature on SIBO and IBS

Title	First author	Study type	Study population	Intervention	Duration	Positive outcome	Negative outcome	Key findings
Profile of rifaximin and its potential in the treatment of irritable bowel syndrome	Natalya Iorio et al. [70]	Phase III, Randomized Double-Blind Placebo-Controlled Trial	1,260 IBS patients, diagnosed by Rome II criteria, without constipation	The study compared rifaximin 550 mg three times a day vs placebo for 14 days. Patients were assessed for an additional 10-week period after treatment	14 days	Patients in the rifaximin group demonstrated adequate improvement of global IBS symptoms	The incidence of adverse events (AEs) was similar among both groups (1.6% vs. 2.4%), and no case of *Clostridium difficile*-associated diarrhea or ischemic colitis was reported	Rifaximin has been shown to improve global IBS symptoms, such as bloating, flatulence, and abdominal pain
Bowel symptoms in patients that receive proton pump inhibitors. Results of a multicenter survey in Mexico	Schmulson M et al. [71]	Double-Blind Placebo-Controlled Multicenter Clinical Trial	1,851 patients	Surveyed patients treated with Proton Pump Inhibitors (PPI), utilizing an ad hoc questionnaire to determine the presence of bowel symptoms and IBS. A total of 77.5% of the patients received treatment for ≤ 6 months and 11.9% for ≥ 1 year	1 year	Post-PPI use: Decreased symptoms (55.9% → 44.1%, P <.005); shift from constipation (63.8%) to diarrhea (56.5%, P <.0001). The highest symptom relief was reported with antibiotics, mainly rifaximin	The dose of PPI was not analyzed. Also, irregular bowel movements began more frequently after PPI use (p < 0.0001)	PPIs are frequently linked to IBS-like symptoms in Mexico, with diarrhea and bloating predominating. Antibiotics, mainly rifaximin, provided the highest satisfaction, suggesting SIBO/dysbiosis as the underlying cause
Effects of Rifaximin on Transit, Permeability, Fecal Microbiome, and Organic Acid Excretion in Irritable Bowel Syndrome	Andrés Acosta et al. [72]	Randomized Double-Blind Placebo-Controlled Trial	Patients with diarrhea-predominant (IBS-D) or mixed IBS (IBS-M; i.e., non-C-IBS), aged 18–75 years	Rifaximin, 550 mg t.i.d., and placebo for 14 days	14 days	Rifaximin was associated with acceleration of ascending colon emptying and overall colonic transit	Rifaximin did not significantly alter total fecal bile acids per g of stool or proportion of individual bile acids or acetate, propionate, or butyrate in stool	In non-constipated IBS without documented SIBO, rifaximin treatment is associated with acceleration of colonic transit time
Rifaximin-Alpha Increases Lactase Activity in Patients with Irritable Bowel Syndrome Without Constipation and Small Intestinal Bacterial Overgrowth	María Fernanda García-Cedillo et al. [41]	Prospective, Open-label, Pilot study	Patients with IBS without constipation (Rome IV), positive for SIBO (lactulose-HBT), low urinary D-xylose, and self-reported lactose intolerance	Rifaximin-alpha 400 mg every 8 h for 14 days	2 weeks of treatment, followed by a 4-week post-treatment evaluation	Symptom improvement: 60% in abdominal pain, 44% in bloating,36% in flatulence, 60% in borborygmi, 72% in stool consistency. Lactose maldigestion (via lactose-HBT) reduced from 88 to 52%	Only 32% achieved SIBO eradication (negative post-treatment lactulose-HBT)	Rifaximin-alpha led to marked symptomatic improvement in IBS patients without constipation, improved lactose digestion, and partially reduced SIBO positivity
Short-course Rifaximin therapy efficacy and lactulose hydrogen breath test in Chinese patients with diarrhea-predominant irritable bowel syndrome	Zhuang X et al. [19]	Randomized Controlled Trial	78 IBS-D patients with positive lactulose hydrogen breath test	Rifaximin 400 mg TID for 10 days	10 days	68% Lactulose Hydrogen Breath Test normalization; 66% had marked symptom relief (GSRS score); improved bloating and discomfort	No placebo control; short follow-up; small sample	Rifaximin is effective in improving symptoms and reducing SIBO in IBS-D patients over a short course
Probiotic Bacteria *Lactobacillus acidophilus* NCFM and *Bifidobacterium lactis* Bi-07 Versus Placebo for the Symptoms of Bloating in Patients With Functional Bowel Disorders: A Double-blind Study	Ringel-Kulka et al. [44]	Prospective, Randomized, double-blind, placebo-controlled clinical trial	60 patients (probiotic, n = 31; placebo, n = 29), 72% females, 84% whites, mean age 37 years	Probiotic bacteria Lactobacillus acidophilus-NCFM and Bifidobacterium lactis Bi-07 (B-LBi07) twice a day (2 × 10^11^ CFU/day) versus placebo	8 weeks	Probiotics led to significant bloating improvement vs placebo at 4 & 8 weeks (P < 0.05); a similar benefit was seen in the IBS subgroup (n = 33)	This study intervention of daily consumption of L-NCFM and B-LBi07 (2⤫10^11^ CFU/d) did not show a significant improvement over placebo on the study’s two primary endpoints: global relief of GI symptoms and satisfaction with treatment	Twice-daily administration of L-NCFM and B-LBi07 was associated with a significant improvement in bloating symptoms among patients with functional bowel disorders (FBDs)
Effect of a Preparation of Four Probiotics on Symptoms of Patients with Irritable Bowel Syndrome: Association with Intestinal Bacterial Overgrowth	Leventogiannis K et al. [43]	Open-label Double-Blind Placebo-Controlled Non-Randomized Clinical Trial	5 patients with IBS and SIBO and 21 patients with IBS without SIBO	Lactolevure® (S. boulardii + 3 probiotics) given BID × 30 days; IBS (Rome III) patients assessed via Irritable Bowel Syndrome – Symptom Severity Scale (IBS-SSS)	30 days	IBS + SIBO patients had a 71.3% IBS score decrease vs 10.6% in non-SIBO (P = 0.017); Satisfaction improved in SIBO, IBS-C, and IBS-D patients; pain decreased in IBS-C regardless of SIBO	The study employed a Non-randomized Study Design, used a small sample size for the SIBO-positive study group, and lacked evaluation of SIBO using a breath test	First prospective study showing superior efficacy of probiotics in IBS + SIBO; notable symptom improvement in both IBS-D and IBS-C subtypes
Treatment of small intestinal bacterial overgrowth in Chilean patients with irritable bowel syndrome: A prospective and comparative study	C von Muhlenbrock w et al. [20]	Prospective Randomized Controlled Trial	97 adult patients (> 18 years) diagnosed with IBS and confirmed SIBO per Rome IV criteria in Chile	Group A received oral rifaximin-α 400 mg twice daily, Group B received ciprofloxacin 500 mg twice daily, and Group C received metronidazole 500 mg three times daily, all for 10 days	15 days of treatment with post-treatment breath testing and symptom reassessments	All three antibiotics improved IBS symptoms significantly Metronidazole showed the highest SIBO eradication rate (79%) Rifaximin had the best tolerability and safety profile	Rifaximin was less effective at eradicating SIBO than metronidazole	Metronidazole was most effective in SIBO eradication, but rifaximin had fewer adverse effects and good symptom control. Ciprofloxacin was comparable to rifaximin but had a higher side effect profile
Effect of *Saccharomyces boulardii* CNCM I-745 on SIBO and Symptoms in IBS-D: A Randomized Open-Label Study	Luis Maria Bustos Fernández et al. [51]	Randomized, Open-label, Controlled Trial	54 patients with IBS-D and confirmed SIBO (via lactulose hydrogen breath test)	Group 1: *Saccharomyces boulardii* CNCM I-745 + dietary advice (Sb + DA) Group 2: Dietary advice (DA) only Treatment duration: 15 days	15 days	Greater symptom reduction in the Sb + DA group: BS Symptom Severity Scale reduced by − 134 vs − 93 in DA group Diarrhea prevalence is lower in Sb + DA (25.9%) vs DA (47.6%)	Only a modest difference in hydrogen excretion between groups 9	*Saccharomyces boulardii* CNCM I-745, when combined with dietary advice, led to better symptom relief, modest SIBO reduction, and favorable modulation of gut microbiota in IBS-D patients
Preferential usage of rifaximin for the treatment of hydrogen-positive small intestinal bacterial overgrowth	Barkin J et al. [23]	Observational Study	53 patients with positive glucose hydrogen breath test (GHBT)	Evaluate patient response to rifaximin [550 mg three times daily for 14 days] for SIBO based on breath test results	14 days	Response rates to rifaximin [550 mg three times daily for 14 days] were 47.4% for hydrogen positivity alone and 80% for both hydrogen and methane positivity	No negative outcomes were reported in the study	Patients with positive breath tests responded well to rifaximin therapy. Rifaximin may prove a highly effective therapy in providing symptom relief from the effects of SIBO
Rifaximin treatment for small intestinal bacterial overgrowth in children with irritable bowel syndrome: a preliminary stud	E.Scarpellini et al [24]	Prospective, open- label, single arm interventional study	50 children with IBS were consecutively enrolled	All subjects underwent lactulose hydrogen/methane breath test to assess SIBO before and one month after the treatment with rifaximin 600 mg daily for one week	1 week	Rifaximin was effective and safe in SIBO treatment, and IBS symptoms improved in childhood	Major limits of this study were the absence of a control group and placebo treatment useful in order to verify preliminary results	A 1-week course of rifaximin 600 mg/day is either effective or safe and tolerable when used for SIBO treatment in children with IBS
Double-Blind Placebo-Controlled Study of Rifaximin and Lactulose Hydrogen Breath Test in Gulf War (GW) Veterans with Irritable Bowel Syndrome	Tuteja A et al. [74]	Double-Blind Placebo-Controlled Study	50 GW Veterans (Predominantly males)with IBS (Rome III)	50 IBS patients randomized to Rifaximin 550 mg BID vs placebo × 2 wks; assessed stool habits, pain, bloating, global improvement; LHBT pre/post-treatment	2 weeks	No positive outcomes were reported in the study	Rifaximin was not associated with significant improvement in global GI symptoms (all P ≥ 0.25) or Quality of Life (all P ≥ 0.26)	Rifaximin was not effective in improving IBS symptoms and quality of life in GW veterans with non-constipated IBS

### Therapeutic strategies for SIBO in IBS

#### Antibiotic therapy

Antibiotics are used as the first line of treatment for managing SIBO in IBS, mainly by acting on the imbalances in the gut microbiota. Reports from many patients show lasting relief of symptoms after the usage of these antibiotics, and they believe the progress is so because these antibiotics work by addressing underlying issues such as SIBO [26]. However, despite the use of antibiotics, the challenge of frequent recurrence of bacterial overgrowth after therapy still persists [29]. Recent consensus guidelines, including those from the American Gastroenterological Association (AGA), recommend a stewardship-based approach, antibiotics should be reserved for patients with well-documented SIBO and clear symptom correlation, while underlying factors, such as motility disorders, structural abnormalities, and dietary triggers, should be identified and corrected first [28].

Among available agents, non-absorbable antibiotics such as rifaximin have demonstrated efficacy with a favorable safety profile, which is aided by its unique chemical and pharmacokinetic characteristics that allow it to exert maximum therapeutic effect with minimal side effects; this upholds it among other antibiotics available for IBS management [30]. Among patients who used Rifaximin, there are noticeable improvements in symptoms, such as bloating, abdominal discomfort, and altered bowel habits, because functionally, rifaximin favorably influences gut microbial populations in IBS patients [31]. There is clinical evidence that shows particular benefit in patients with IBS-D, as it alleviates global symptoms, reduces abdominal distension, and improves overall quality of life [29]. The drug acts on gut microbiomes by temporarily reducing the microbial diversity, especially by lowering populations of bacterial families that are associated with IBS symptoms, such as *Enterobacteriaceae*, *Verrucomicrobiaceae*, and *Peptostreptococcaceae* [32]. Sustained symptom improvement in multiple studies showed the use of a standard dosing regimen of 550 mg three times daily for 14 days [29]. Rifaximin also holds FDA approval for its use in IBS-D as it has caused reductions in specific microbial groups like *Clostridiales* [31]. However, these figures must be interpreted cautiously. Placebo response rates in IBS-D trials are consistently high (20–40%), meaning the absolute benefit of rifaximin over placebo is considerably more modest than response rates alone suggest. Efficacy also varies substantially across trials due to differences in patient selection, SIBO diagnostic criteria, and definitions of treatment response. Most critically, breath test normalization and symptom improvement are frequently dissociated; rifaximin often improves symptoms without achieving SIBO eradication, raising fundamental questions about whether its benefit operates through SIBO clearance or through broader immunomodulatory and microbiome-stabilizing mechanisms independent of SIBO status [24, 29]. Symptom recurrence after therapy has been reported in up to 40–60% of cases, sometimes prompting repeated or maintenance courses [29]. When such retreatment is considered, clinicians should balance potential benefits with risks, such as *C*. *difficile* infection, microbial dysbiosis, and emerging antimicrobial resistance, as emphasized in AGA best-practice advice.

For the treatment of IBS-C, there are options ranging from laxatives and probiotics to antibiotics and centrally acting medications. In patients with IBS-C, slower gut transit occurs due to methane-producing microbes known as methanogens. Laxatives help disrupt this favorable environment for methanogens by increasing the bowel movement frequency, thus offering symptomatic relief. In methane-positive IBS-C patients, non-absorbable antibiotics like rifaximin and neomycin exhibit particular efficacy. Clinical evidence indicates that there are more noticeable improvements in patients with constipation that show methane production when treated with these antibiotics in comparison to those without detectable methane production. Rifaximin improves colonic transit and reduces methane concentrations during testing, while neomycin is known to normalize lactulose breath test results. Additionally, combining rifaximin and neomycin has proven to offer better control of complicated symptoms like bloating and straining than neomycin alone [11]. It is important to note, however, that rifaximin monotherapy shows limited efficacy in methane-positive patients, and response rates in IBS-C overall are less robust and less consistently reported than in IBS-D, reflecting the greater therapeutic challenge posed by methanogen-driven pathophysiology [21, 33]. Most methane-positive IBS-C patients treated with antibiotics and/or dietary fiber report symptomatic relief and improved follow-up breath test outcomes [34].

Latest research is now focusing on designing drugs that solely target methane-producing microbiomes without manifesting any other side effects. Statin-derived lovastatin is one such drug that does not exhibit recognized concerns like antibiotic resistance with long-term use [11]. Certain fluoroquinolones, like ciprofloxacin, cause a reduction in the number of gram-negative organisms in the gut due to their bactericidal activity, which is often accompanied by increased levels of gram-positive bacteria, such as bifidobacteria and lactobacilli. However, the role of rifaximin and neomycin is more established and pronounced than fluoroquinolones in IBS treatment [35].

Metronidazole stands out as an antibiotic for SIBO with the highest eradication in comparison to other available antibiotic options for SIBO. Nonetheless, it should be used with caution due to its broader antimicrobial spectrum and higher risk of adverse events [20]. On the other hand, rifaximin contributes by establishing more reliable symptomatic improvement alongside a favorable safety profile. SIBO has a tendency to recur, which is a challenge in its management, often necessitating repeated or maintenance courses of therapy. Of the agents studied, rifaximin has consistently delivered effective results both for initial treatment and for managing symptom recurrences [29]. To maintain symptom remission across various IBS subtypes, medications like tegaserod have repeatedly proved their potential, be that as it may, their availability remains restricted in some regions [26].

In the management of targeted subgroups like methane-positive IBS-C and IBS-D, while rifaximin and neomycin continue to play a crucial role, probiotics have also proved to be adjunctive treatment in IBS. Although findings from across studies remain inconsistent, certain formulations of probiotics have demonstrated benefits in alleviating symptoms [15]. Rifaximin stands out as one of the safest and most effective agents currently available [36]. Patients with IBS possess the characteristic of recurrent bacterial overgrowth, which has a pronounced influence on the development of personalized treatment approaches and combination therapies. Ongoing research into newer options, such as resistance-sparing agents, probiotic strategies, and diet-based interventions, has also become more prominent due to recurrent bacterial overgrowth [37]. Usage of these evolving therapeutic strategies (Table 3) may eventually improve long-term outcomes for IBS patients. However, developing an advantageous approach for managing IBS requires extended follow-up studies, knowledge on pharmacological management, and greater clarity on the mechanism of action through which these therapies exert their effects [38].

**Table 3:** Summary of Antibiotic Therapy used for Management of SIBO in IBS

Drug	Dose	Duration	Efficacy	Common side effects	Relapse rate
Rifaximin	400–550 mg (about half the weight of a small paper clip), 2–3 ×/day	10–14 days	32–68% SIBO eradication; symptom relief up to 66% [19, 26, 29]	Well-tolerated; mild GI upset in some	Variable; up to 44–60% reported
Metronidazole	500 mg, 3 ×/day	10 days	Highest eradication rate (79%) [26]	Nausea, metallic taste, potential for neuropathy	Not consistently reported
Ciprofloxacin	500 mg, 2 ×/day	10 days	Comparable to rifaximin in symptom relief [26]	GI upset, risk of tendonitis, dysbiosis	Not reported
Norfloxacin	800 mg/day	10 days	100% SIBO eradication in a small sample [25]	Not detailed; similar to fluoroquinolones	Limited data (small sample)
Herbal Therapy	Varies (multiple agents)	4 weeks	46% eradication [27]; better than rifaximin in one study	Mild GI effects, generally well tolerated	Not reported

#### Dietary therapy

Addressing dietary habits along with antimicrobial treatment of SIBO often results in suboptimal outcomes. Only focusing on antibiotic treatment may sometimes even worsen gastrointestinal health. In order to optimally manage SIBO, altering dietary practices plays a critical role. Among the dietary strategies are inclusion of low fermentable oligosaccharides, disaccharides, monosaccharides, and polyols (FODMAPs) diets, single-strain probiotics, fiber supplementation, and mindful eating habits. Tailoring the dietary intake of individuals with IBS with these has shown appreciable benefit [39].

**Fig. 3:**
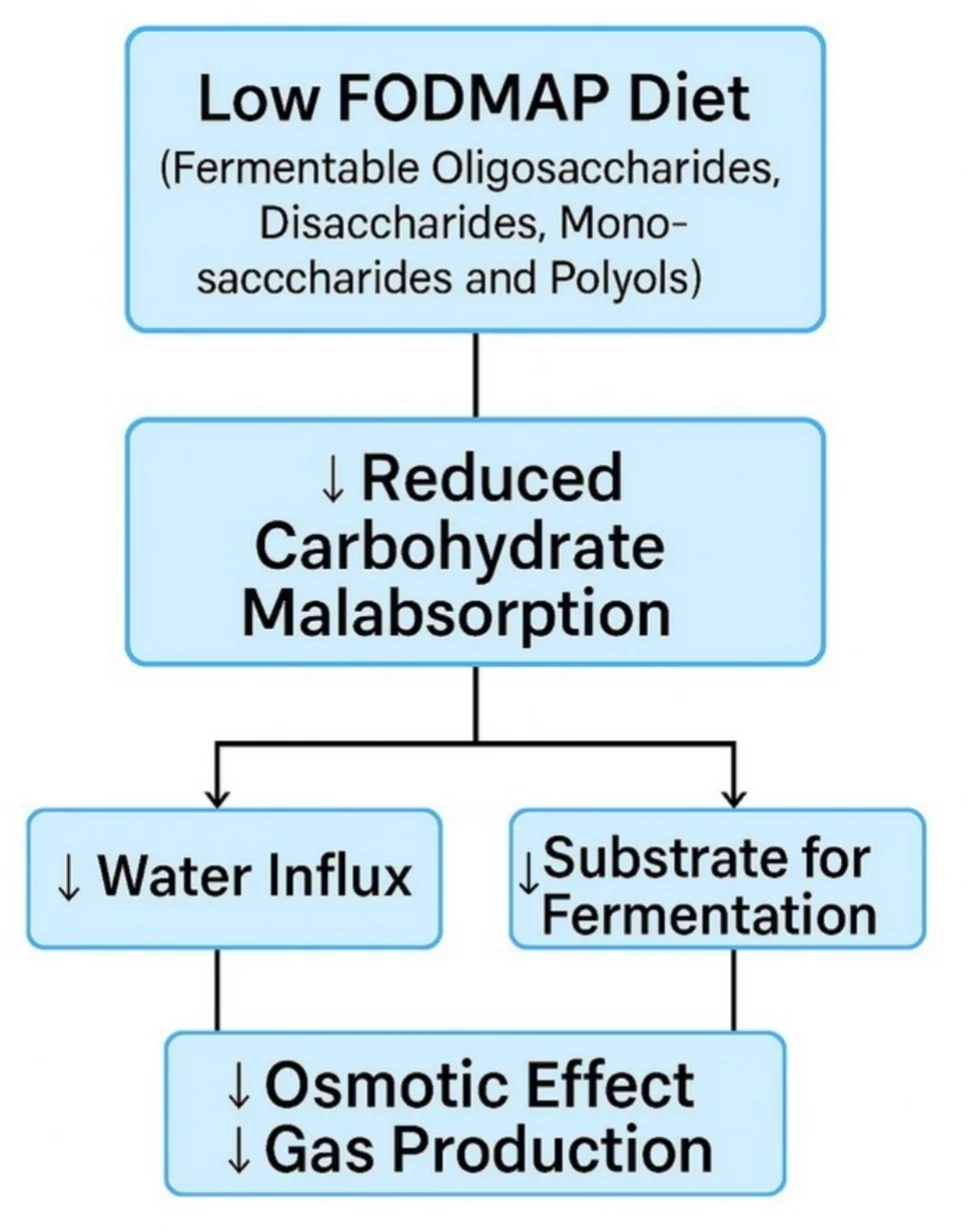
Mechanism of Symptom Reduction with a Low-FODMAP Diet [39]. This figure illustrates how a low-FODMAP diet reduces carbohydrate malabsorption, leading to decreased water influx and substrate for fermentation, thereby minimizing osmotic effects and gas production

FODMAPs are a group of short-chain carbohydrates that are predominantly found in various legumes, grains, and some fruits and vegetables. They exert an osmotic effect by drawing water into the small intestine, promoting fermentation by the gut microbiome and increasing colonic gas production, which triggers pronounced symptoms in viscerally hypersensitive IBS patients [40][Fig. 3]. A low-FODMAP diet, administered for a limited and defined period, can provide meaningful short-term relief from abdominal discomfort, bloating, and altered bowel habits, with symptomatic response rates reported in approximately 50–75% of IBS patients across trials. However, it is critical to distinguish this short-term symptomatic benefit from the long-term microbiome consequences of sustained dietary restriction. Prolonged adherence to a low-FODMAP diet has been consistently associated with significant reductions in total bacterial load, and particularly in beneficial, anti-inflammatory taxa, such as *Bifidobacterium* and *Faecalibacterium prausnitzii*. Organisms whose depletion may worsen the underlying dysbiosis are already present in SIBO-associated IBS [39]. This raises an important clinical concern: while the diet suppresses fermentable substrate availability and thus reduces symptom-generating gas production, it may simultaneously deprive the gut of the prebiotic substrates necessary for maintaining a diverse and resilient microbiome. For this reason, the low-FODMAP diet should be employed as a short-term intervention with structured reintroduction phases, rather than as an indefinite dietary pattern, particularly in patients with pre-existing SIBO where microbiome diversity is already compromised.

Probiotic therapy represents a complementary strategy in IBS-SIBO management, though the evidence base is notably heterogeneous and strain-dependent, and broad generalizations about benefit are not supported by the current literature (Table 4) [39]. Among the better-studied examples, *Saccharomyces boulardii* combined with dietary advice produced greater symptom improvement than dietary advice alone with modest SIBO reduction (41% vs. 29%), and a multistrain preparation achieved adequate symptom relief in 48% of patients versus 12% on placebo over 8 weeks [41, 42]. However, a substantial proportion of patients do not respond to probiotic therapy alone, most trials are short [4–8 weeks], and evidence regarding long-term microbiome reconstitution or durability of symptom relief following probiotic cessation remains limited [42–44]. Probiotics are therefore best considered an adjunct to antibiotic and dietary therapy rather than a standalone intervention, with optimal sequencing and duration requiring further study [37].

**Table 4:** Summary of probiotic therapy used for management of SIBO and IBS

Probiotic preparation	Composition	Duration	Efficacy	Common side effects
*Saccharomyces boulardii* CNCM I-745	*S. boulardii* + Dietary Advice [41]	15 days	Greater symptom improvement vs. dietary advice alone; modest SIBO reduction (41% vs 29%)	Well tolerated
Lactolevure®	*S. boulardii*, *B. lactis*, *L. acidophilus*, *L. plantarum* [43]	30 days	71.3% symptom score reduction in IBS with SIBO; higher satisfaction vs IBS without SIBO	No significant reported
Multistrain Probiotic Mixture	*L. acidophilus, L. plantarum, L. rhamnosus, B. breve, B. lactis, B. longum, S. thermophilus* [42]	8 weeks	48% achieved adequate symptom relief vs. 12% placebo; improved stool consistency; microbiota stabilization	Mild bloating occasionally
L-NCFM + B-LBi07	*Lactobacillus acidophilus* NCFM + *Bifidobacterium lactis* Bi-07 [44]	8 weeks	Improved bloating severity significantly vs placebo at 4 and 8 weeks	No significant reported
Multistrain Probiotic	Not specified (study mentions beneficial/harmful bacteria ratio improvement) [47]	8 weeks	Decreased harmful bacteria, improved GI symptoms, decreased SIBO prevalence	Not specified

**Fig. 4:**
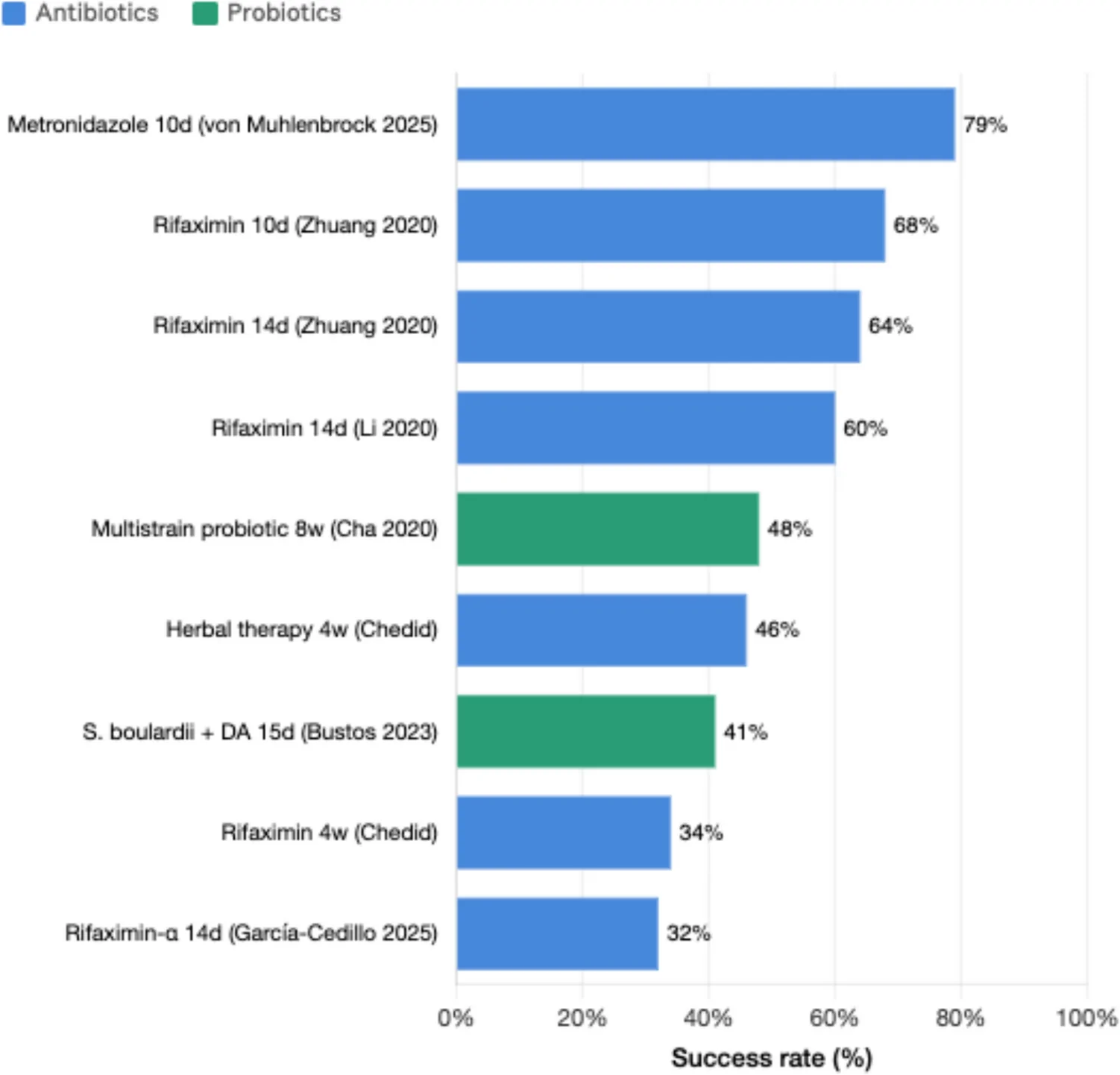
SIBO eradication and clinical response rates for antibiotic and probiotic interventions [19, 20, 27, 31, 41, 42, 51, 73]. This figure illustrates SIBO eradication and clinical response rates for antibiotic and probiotic interventions. Success rates represent either SIBO eradication (negative post-treatment breath test) or adequate clinical response (≥ 50% symptom improvement) as reported in published studies. Metronidazole demonstrated the highest eradication rate (79%), while herbal therapy achieved comparable success to rifaximin with a favorable tolerability profile

Supplementation with specific soluble dietary fiber supports the growth of beneficial prebiotic bacteria and may help maintain gut microbiome balance in patients with IBS and SIBO. Additional dietary triggers, including vitamin deficiencies (B12, A, D, E) and mineral deficiencies (iron, calcium) [45], amine-rich foods [46], excess caffeine, and certain legumes, can exacerbate IBS symptoms and should be considered in individualized dietary planning [2, 46]. Clinicians’ attention to self-reported food intolerances and personalized dietary counseling remains central to effective management. Ultimately, a well-tailored combination of antibiotic therapy with individualized dietary intervention, accounting for both short-term symptom control and long-term microbiome preservation, represents the most balanced and evidence-informed approach to managing IBS with concurrent SIBO [37] [Fig. 4].

#### Clinical presentation and treatment response across IBS subtypes

The complexity of IBS calls for a tailored approach when it comes to understanding how SIBO presents and how patients respond to treatment. Each subtype of IBS has its unique clinical features, connections to SIBO, and responses to therapy, which help shape more effective management strategies.

#### IBS-D (diarrhea-predominant IBS) and SIBO

IBS-D is the most researched subtype in relation to SIBO, showing a particularly strong connection to hydrogen-dominant bacterial overgrowth. Patients often experience frequent urgent and loose stools along with bloating, abdominal pain, and gas [19, 41, 48]. A rapid transit time in the intestines can lead to the colonization of the distal small bowel by fermentative bacteria, resulting in excess hydrogen production and osmotic diarrhea [2]. This could be diagnosed by breath tests, which usually reveal elevated hydrogen levels within 90 min after consuming a substrate, typically lactulose or glucose. The key indicator is elevated hydrogen (H₂) levels, often exceeding 20 parts per million above baseline [37].

Treatment is often a multimodal approach. Rifaximin has shown remarkable effectiveness in treating IBS-D, with several randomized controlled trials indicating significant symptom relief [2, 22, 49]. García-Cedillo et al. (2025) found a 60% improvement in abdominal pain, a 44% reduction in bloating, and a 72% enhancement in stool consistency after rifaximin treatment [41]. Zhuang X (2020) reported a 68% normalization rate in breath tests and a 66% notable symptom relief among Chinese IBS-D patients [19]. For those with hydrogen-positive SIBO, response rates with rifaximin alone are around 47.4%, which jumps to 80% when both hydrogen and methane are present [50]. Along with dietary intervention, such as implementing a low-FODMAP diet, has shown particularly strong benefits for IBS-D patients with SIBO, and elemental and semi-elemental diets lead to quick symptom relief, likely due to a decrease in available fermentable substrates. Along with probiotic therapy, multistrain probiotics have shown some pretty impressive benefits. For instance, Lee S found that beneficial bacteria increased from 41.2% to 53.7%, while harmful bacteria dropped from 13.0% to 4.7%. When *Saccharomyces boulardii* was paired with dietary advice, it led to a greater reduction in symptoms compared to just dietary advice on its own [47, 51].

A clinically relevant subset within IBS-D is PI-IBS, where SIBO may arise as a direct consequence of post-infectious gut dysbiosis, mucosal damage, and impaired motility. In these patients, the overlap between PI-IBS and SIBO is particularly pronounced, and targeted antibiotic therapy with rifaximin is a reasonable first-line approach, especially in those with positive breath tests. Notably, standard rifaximin trials have shown limited efficacy in PI-IBS specifically, attributed partly to suboptimal dosing and diagnostic limitations rather than true antibiotic failure [9]. Emerging evidence suggests that microbiome-profiling-guided therapy may outperform standard treatment in this subgroup, reinforcing the value of precision approaches when managing PI-IBS with concurrent SIBO [10].

#### IBS-C (constipation-predominant IBS) and SIBO

Patients with IBS-C often have methane-producing archaea, which are now recognized as part of Intestinal Methanogen Overgrowth (IMO). These organisms can slow down the colonic transit, which leads to constipation. People typically experience hard or lumpy or bulky stools, straining, a feeling of incomplete voiding, tenesmus, and significant bloating. The delayed transit time leads to increased production of methane in the gut, which makes constipation symptoms even worse. This is diagnosed by elevated methane (CH₄) levels (≥ 10 ppm) detected through breath testing, which are strongly associated with IBS-C [21, 52].

Treatment includes antibiotic therapy. Combining rifaximin with neomycin has shown better results for those dealing with methane-producing SIBO. However, rifaximin alone doesn’t work very well for those with methane positivity. Harvie RM (2019) found that 22% of patients tested positive for methane, which was strongly linked to constipation symptoms. Treatment plans often need to be extended or repeated because methane producers tend to resist standard antibiotic treatments [21]. The Dietary Intervention Response for a low-FODMAP diet can vary quite a bit between IBS-C and IBS-D. Managing fiber intake is crucial, with soluble fiber generally being better tolerated than insoluble fiber. Due to the intricate relationship between fiber consumption and methane production, personalized dietary strategies are key. Along with the one mentioned above, the emerging therapies such as targeted probiotics are showing potential, especially those that might compete with methane-producing methanogens [43], and prokinetic agents could offer additional benefits by tackling the underlying motility issues. It's important to note that methane levels not only link to constipation but also indicate a poor response to rifaximin monotherapy, highlighting the necessity for dual antibiotic treatments in this particular subtype [21, 52].

#### IBS-M (mixed-type IBS) and SIBO

Patients with IBS-M experience a complex alternating pattern of diarrhea and constipation and have > 25% of their bowel movements associated with Bristol type 1 or 2 (hard/lumpy) and Bristol type 6–7 (loose/watery) within the same reporting period, meeting the Rome IV diagnostic threshold [53]. As the symptoms fluctuate over time, a single-visit diagnosis is insufficient. The pathophysiology of IBS-M is multifactorial and particularly complex, driven by varying hydrogen and methane levels, overlapping microbial profiles, and erratic motility patterns [54]. Soldi et al. found that in patients with IBS-M, *Faecalibacterium prausnitzii* was reduced; following treatment with rifaximin, it increased, suggesting that it could have some specificity toward IBS-M [55].

The GBA contributes meaningfully to disease perpetuation in IBS-M. Notably, in a study conducted by Kibune-Nagasako et al., IBS-M patients showed higher anxiety scores than IBS-C patients, as well as a higher prevalence of comorbidities, including GERD, functional dyspepsia, chronic headache, and fibromyalgia, reflecting the broader systemic burden of the mixed subtype [56]. Treatment outcomes in IBS-M are generally less satisfactory and predictable than those observed in IBS-D or IBS-C, reflecting the heterogeneity of underlying mechanisms [20]. Antibiotic combination therapy is more likely to yield favorable outcomes than monotherapy. Low et al. demonstrated that of subjects receiving rifaximin and neomycin combination therapy, 85% had a clinical response, compared with 56% in the rifaximin-only group, and combination therapy achieved 87% methane eradication versus only 28% with rifaximin alone [57]. Response rates vary considerably based on which microbial population is predominant at the time of intervention, reinforcing the value of serial breath hydrogen and methane testing at four-to-six-week intervals to guide therapeutic recalibration as the gas profile evolves following partial treatment [20, 48, 52]. One of the unique pharmacological challenges in IBS-M is the antagonistic relationship between phase-specific treatments: prokinetic agents used during constipation-dominant phases risk exacerbating diarrhea, while antispasmodics and antimotility agents used during diarrhea-dominant phases may precipitate or worsen constipation. These agents should therefore be sequenced according to the patient's current symptom predominance. A multimodal approach combining antibiotics with targeted dietary modification consistently outperforms either strategy alone [43, 48]. Dietary management in IBS-M requires a dynamic, phased approach that accounts for the shifting nature of symptoms. Implementing a low-FODMAP diet might require changes depending on which symptoms are the most prominent. The timing of the dietary changes also has a key role to play for achieving a better outcome [58].

Clinicians should also be aware that IBS-M represents a phenotypically unstable subtype; approximately 30% of IBS patients undergo subtype transitions within six months, and IBS-M appears to be the least stable phenotype, which underscores the necessity of dynamic, reassessment-driven management rather than fixed long-term treatment plans [59].

Thus, understanding the different clinical presentations and treatment responses among IBS subtypes highlights the need for personalized strategies in managing SIBO. This approach moves us away from the age-old generic treatment plans and toward more targeted and subtype-specific strategies [Table 5].

**Table 5:** Comparison of SIBO features and treatment by IBS Subtype

IBS subtype	Common SIBO type	Key symptoms	Preferred diagnostics	Treatment options
IBS-D	Hydrogen-dominant SIBO	Diarrhea, urgency, bloating, cramping	LHBT, GBT (high H2)	Rifaximin, low-FODMAP diet, and probiotics (e.g., boulardii)
IBS-C	Methane-Predominant SIBO	Constipation, straining, bloating	CH₄ ≥ 10 ppm on breath test	Rifaximin + Neomycin fiber modulation; microbiota-targeted therapy
IBS-M	Mixed	Alternating diarrhea and constipation	Variable; repeat or dual testing	Tailored antibiotics; FODMAP diet

In summary, antibiotic therapy for SIBO in IBS should follow a stewardship framework: confirm diagnosis before treatment, select the narrowest effective agent, limit duration to 10–14 days, avoid empiric chronic or maintenance use, and reassess underlying causes and nutritional status before repeating courses. Combining antibiotics with dietary modification and probiotic strategies may help maintain remission while minimizing antimicrobial exposure and resistance risk.

### Clinical implications

The therapeutic paradox in IBS management lies in the observation that while broad-spectrum systemic antibiotics have been implicated in the pathogenesis of IBS through microbiota disruption, a non-systemic antibiotic like rifaximin is one of the most effective pharmacologic treatments for IBS with diarrhea (IBS-D) [60]. An overall success rate of 70.8% for rifaximin therapy in eradicating SIBO was demonstrated by Gatta el al., in a systematic review and meta-analysis, however the findings demonstrated high heterogeneity (I^2^ = 89.4%) and thus must be interpreted with caution [61].

SIBO is unlikely to be the primary cause of IBS as iterated earlier due to no consistent, dose–response relationship or temporal correlation [61]. These findings suggest that SIBO may act as a *disease modifier* within the broader IBS spectrum rather than a singular cause. Short, 2-week courses of rifaximin have shown efficacy and safety in randomized control trials [62]. The mechanism remains unclear, although evidence points toward modulation rather than eradication of gut bacteria, particularly treatment of SIBO and stabilization of gut microbial communities.

Given the cost and potential side effects, several non-pharmacological treatments have gained attention. One such approach is an elemental diet. Which consists of predigested micronutrients absorbed predominantly in the proximal small intestine, reducing the nutrient availability to bacteria in the distal portion of small bowel. In a retrospective review, 124 patients with SIBO received an elemental diet for a duration of 14 days, the cumulative symptomatic response rate achieved was 85%, with normalization of breath tests in most responders at 1-month follow-up. However, tolerability and adherence remain challenging [63]. Similarly, low-FODMAP diets, which limit fermentable carbohydrates, have shown robust efficacy in IBS through reductions in luminal osmotic load and gas production, although direct benefits in SIBO are less well established [63].

The goal of antibiotic therapy in SIBO is not complete eradication but modulation. In vitro susceptibility testing in principle, could inform antibiotic selection, but is generally impractical due to coexistence of diverse bacterial species with variable sensitivities. Consequently, antibiotic treatment remains largely empirical, with limited comparative data to support the superiority of any single regimen. Typically, a single 7–10 day antibiotic course leads to symptomatic improvement lasting several months in about 46%–90% of SIBO patients and normalizes breath test results in roughly 20%–75% of cases. In practice, an effective course should target both aerobic and anaerobic enteric bacteria. Repeated or maintenance antibiotic cycles should be approached with caution, as robust data on relapse rates, adverse events, and resistance patterns are lacking [64, 65].

From a stewardship perspective, management decisions should be individualized and guided by AGA and ACG best-practice recommendations, emphasizing identification and treatment of underlying causes (such as motility disorders, anatomical abnormalities, or chronic PPI use), correction of nutritional deficiencies, and prioritization of non-pharmacologic measures (e.g., dietary modification) before initiating repeat antibiotic therapy. Clinicians should also weigh the potential risks of antibiotic-associated diarrhea, *Clostridioides difficile* infection, intolerance, and financial cost against the expected benefits. [65, 66].

### Limitations in current literature and future directions

Despite several studies that maintain a relationship between SIBO and IBS, significant limitations exist. Most trials inappropriately overestimate IBS-D, with underrepresentation of subtypes IBS-C and IBS-M, which has corresponding generalizability limitations [20, 41]. Heterogeneity is high in diagnostic testing, particularly breath tests. In the absence of standardization in substrates (lactulose vs. glucose), cut-offs, and interpretation criteria, findings are inconsistent [8, 21]. Invasive methods like selective small bowel aspirate cultures, though more sensitive [25], are impractical for use in the clinical setting. Regimens differ in drug usage, dose, and duration, with minimal follow-up beyond the long term. Outcome is most commonly reported at 2–4 weeks, yielding little information regarding relapse rates or long-term effectiveness.

Future research needs to focus on subtype-specific RCTs with standardized diagnostics and a minimum 6-month follow-up to establish durability and relapse. Antibiotic stewardship and prudent use of PPI therapies need to be emphasized to prevent the development of resistant and refractory microbiota strains in SIBO pathogenesis, respectively. More novel modalities like hydrogen sulfide testing and gut microbiota profiling, which could potentially culminate in fecal microbiota transplantation (FMT) for recalcitrant SIBO, are promising in guiding therapy [31]. Novel approaches like targeted probiotics, herbal therapies [27], and dietary approaches (e.g., low-FODMAP, low-fat) [22] support a shift toward precision, microbiome-based treatment.

## Conclusion

Small bowel bacterial overgrowth is increasingly being recognized as an associated factor in a considerable majority of irritable bowel syndrome cases. The occurrence of overlapping SIBO and IBS symptomatology like bloating, abdominal discomfort, and altered bowel habits reinforces its role in symptom formation and maintenance of disease. It is particularly significant when objective diagnostic methods such as jejunal aspirate culture or hydrogen/methane breath tests are employed [11, 25]. IBS-D possesses the most evidence for the effectiveness of SIBO-directed treatments among the IBS subtypes. Several studies show that rifaximin significantly reduces symptoms in IBS-D patients [4, 48]. When combined with dietary interventions like the low-FODMAP diet, which lowers fermented substrates for bacterial metabolism, this benefit is frequently amplified [1, 5]. These studies document significant clinical outcomes, such as decreases in bloating, irregular stool, and abdominal pain, as well as objective improvements, such as normalization of breath tests [5].

On the other hand, a more complex, multimodal strategy is needed to treat IBS-C and IBS-M. The standard rifaximin monotherapy is not as efficient in treating intestinal methanogen overgrowth (IMO) or methane-predominant SIBO, which are frequently linked to IBS-C. Combination treatments, like rifaximin and neomycin, have demonstrated increased effectiveness in lowering methane levels and symptoms associated with constipation [26, 67]. With varying profiles of symptoms and mixed production of gas, IBS-M is still even more variable, requiring tailored treatment plans that are informed by symptom predominance, frequent diagnostic testing, and occasionally behavioral therapies [68, 69]. Preliminary evidence suggests that combining antibiotics with dietary modification may yield greater improvement than either intervention alone; for example, rifaximin followed by probiotic or diet-based maintenance [1, 69]. However, these findings remain exploratory. Future research should prioritize subtype-specific randomized controlled trials using standardized diagnostic methods and ≥ 6–12-month follow-up to evaluate sustained efficacy and relapse prevention. Such studies could help clarify how best to integrate microbiota-targeted and behavioral therapies in IBS management.

However, there are still a lot of important gaps in spite of promising results. IBS-D has received the majority of clinical trials' attention, while IBS-C and IBS-M have gotten significantly less. Additionally, universal treatment approaches are hindered by variability in antibiotic regimens, heterogeneity of diagnostic methods, and limited long-term data [8, 24]. For this reason, increased subtype-specific research is needed that employs longer follow-up, standardized diagnostics, and precision medicine paradigms that can incorporate adjunctive therapies, methane/hydrogen stratification, and microbiota profiling. Finally, SIBO may contribute to symptoms in a subset of IBS patients most consistently IBS-D yet causality and long-term treatment durability remain uncertain. Optimal treatment requires an individualized and multidisciplinary approach. Improving long-term results and the standard of life among this complicated patient population will require targeted research, dietary and antibiotic strategy integration, and therapy customization based on IBS subtype (469). In conclusion, while bacteria may contribute to some IBS-associated symptoms, they cannot completely account for the condition. A direct causal link between IBS and SIBO should therefore not be assumed.

The absence of a consistent dose–response relationship between bacterial load and symptom severity, the lack of temporal data establishing SIBO as antecedent to IBS onset, and the persistent dissociation between breath test normalization and symptom resolution collectively argue against a universal causal role [54]. Resolving this question will require validated small bowel sampling, longitudinal microbiome profiling, and subtype-stratified trial designs.

## Data Availability

Data sharing is not applicable to this article, as no new data were created or analyzed in this study.

## Declarations

### Large language model (LLM)—generative AI use statement

We have employed a Large Language Model (ChatGPT, OpenAI, Claude) to refine the English language and improve readability. This process focused solely on improving the text's clarity and style, without generating or adding any new information to the content.
